# The effect of positive thinking training on hope and adherence to treatment in hemodialysis patients: a randomized controlled trial

**DOI:** 10.1186/s40359-023-01036-2

**Published:** 2023-01-09

**Authors:** Fatemeh Sabouri, Masoume Rambod, Zahra Khademian

**Affiliations:** 1 Student Research Committee, School of Nursing and Midwifery, Shiraz University of Medical Sciences, Shiraz, Iran; 2 Department of Nursing, Community Based Psychiatric Care Research Center, School of Nursing and Midwifery, Shiraz University of Medical Sciences, Shiraz, Iran; 3 Department of Nursing, School of Nursing and Midwifery, Shiraz University of Medical Sciences, Shiraz, Iran

**Keywords:** Education, Hemodialysis units, Hospital, Psychology, Positive, Renal dialysis, Treatment adherence and compliance

## Abstract

**Background:**

Patients undergoing hemodialysis are exposed to psychological problems, such as despair, which in turn can be a trigger for them to abandon the treatment process. This study aimed to determine the effect of positive thinking training on hope and adherence to treatment in hemodialysis patients.

**Methods:**

This randomized controlled trial was performed on 80 hemodialysis patients referred to two hemodialysis centers in Shiraz, Iran. They were randomly divided into an intervention and a control group. Eight sessions of positive thinking skills training carried out individually on the patients' bedsides. The primary and secondary outcomes were hope and adherence to treatment, respectively. The data were collected using Snyder Hope Questionnaire, End-Stage Renal Disease Adherence Questionnaire, laboratory tests, and weight measurements. Data were analyzed by Chi-square and Paired and Independent T-test using SPSS software version 18.

**Results:**

After the intervention, the mean score of hope was significantly higher in the intervention group (42.1 ± 6.1) than in the control group (38.7 ± 6.5) (*p* = 0.024). Moreover, after the intervention, the mean score of adherence to treatment was significantly higher in the intervention group (1070.2 ± 80.1) compared to the control group (1018.4 ± 105.3) (*p* = 0.019). In addition, blood urea nitrogen, phosphate and inter-dialytic weight gain were lower in the intervention group compared to the control group after the intervention.

**Conclusions:**

The findings showed that positive thinking interventions could lead to improvement in hope and adherence to treatment in hemodialysis patients. Positive thinking training could be used in caring of hemodialysis patients to improve their hope and adherence to treatment.

*Trial registration* RCT Registry: Iranian Registry of Clinical Trials; RCT registration number: IRCT20180915041044N1; Registration date: 19/12/2018.

## Background

End stage renal disease (ESRD) is one of the most common chronic diseases worldwide [1, 2]. In Iran, the disease has an annual growth rate of approximately 4–5%. Given the country's 1.2% population growth, treating the disease is a challenge, both medically and economically, for the country. Moreover, Iran is among the countries with the lowest incidence of the disease. Hemodialysis is the most common renal replacement therapy whose main purpose is bringing the life of patients with renal diseases closer to the normal status [1, 3, 4].

ESRD and dialysis make major changes to the patients' normal lifestyles. In addition to the various physical effects, it has numerous psychosocial consequences and results in dramatic changes in their daily lives [5, 6]. Consequences like frequent hospitalization, need for complex treatment regimens, skin and mucosal manifestations, and fear of disability and death lead to a difficult life for patients and their families [7]. These patients are often worried about their unpredictable future and may suffer from depression and hopelessness [8, 9]. Meanwhile, hope is a factor that can protect people from life's stressful events. Hope is a process in which one sets goals, creates solutions to achieve them, and creates the motivation to execute and maintain them along the way[10]. Therefore, hope is considered an effective factor in coping with chronic diseases [11].

Adherence to treatment regimens is of great importance in patients with chronic diseases [12]. Hemodialysis can bring about changes in the lifestyle, health, and roles of patients [13]. Adherence is essential for having high-efficiency hemodialysis, achieving optimal therapeutic outcomes, and reducing the complications, disability, and mortality [14]. Evidence has suggested that non-adherence to treatment was relatively high among hemodialysis patients [15]. The findings of a descriptive-analytic study in Iran showed that 77% of the hemodialysis patients had moderate treatment adherence and their adherence was positively related with their quality of life [16]. A meta-analysis showed that various interventions including educational, counseling, psychological, and behavioral interventions have reported moderate effects on some outcomes of treatment adherence in patients with ESRD [17].

In the recent years, psychologists have been emphasized on positive thinking and tried to focus people on their capacities and capabilities despite the illness. The ultimate goal of this approach is to identify the components and methods that promote humans' health and happiness [18–20]. According to Quilliam, positive thinking means having the appropriate inner balance, keeping calm when confronted with problems, and using helpful and effective ways in challenging situations. Positive thinking does not mean not confronting problems, but it is about taking a proper approach without breaking down when faced with problems [18]. People who think positively cope better with psychological stress and build better social support networks around them by using more effective strategies, such as reassessment and problem-solving. They also follow healthier lifestyles that protect them from diseases. Even if such people get sick, they will perform better on medical advice. They follow these recommendations with appropriate behavioral patterns that accelerate recovery [19]. Positive thinking interventions have shown positive effects on individuals' physical and psychological outcomes [21, 22]. Previous studies have shown that positive thinking training improved the quality of life of hemodialysis patients [23], resilience and quality of life of patients with cancer [24], and hope and sleep quality of patients with thalassemia major [22].

Hemodialysis patients usually have low hope scores [25]. In addition, these patients are often frustrated with their treatment due to the chronicity of their illness and may abandon their treatment [15, 16]. Nurses can play an important role in promoting adherence to treatment among hemodialysis patients and help improve their adherence by establishing a strong and supportive relationship with them [26]. Although psychological interventions are widespread today, the effectiveness of positive thinking interventions in hope and treatment adherence has been less studied in this group of patients. Therefore, given the high likelihood of hopelessness and discontinuation of treatment in patients with ESRD, the present study aimed to determine the effect of positive thinking training on hope and adherence to treatment amongst hemodialysis patients.

## Methods

### Design and participants

This randomized controlled trial with the pre/post-test control design was performed in two hemodialysis centers located in two hospitals in Shiraz, Iran from February to May 2019. We have complied with CONSORT standards in the report of this trial. At the first, ethical approval was obtained from the Ethics Committee of Shiraz University of Medical Sciences, Shiraz, Iran.

Based on a study aimed to determine the effectiveness of motivational interviewing in depression and hope in hemodialysis patients [9], using the Med-Calc software, and considering β = 90%, α = 5%, µ1 = 26.2, σ1 = 3.42, µ2 = 23, and σ2 = 3.79 a 56-subject sample size was determined for the study. Considering the 20% probability of loss, the total sample size was estimated as 80 participants (40 participants in each group).

**Fig. 1:**
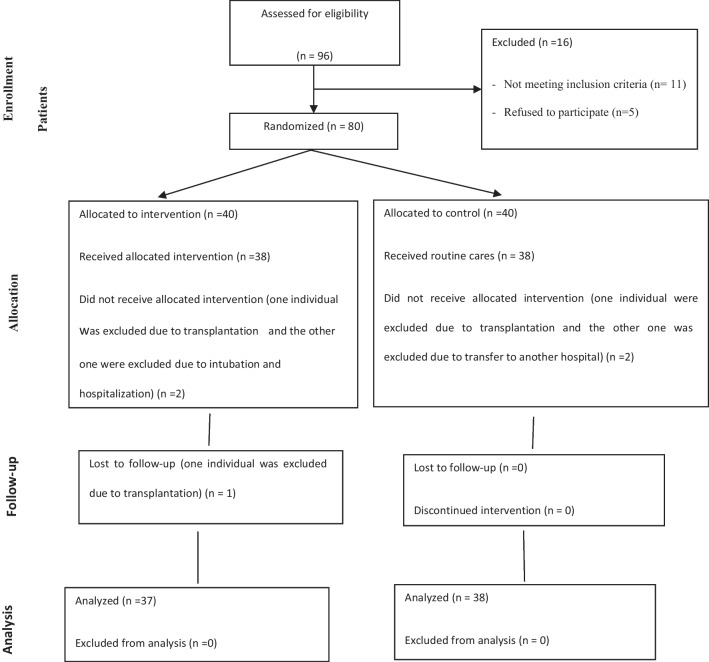
Consort diagram of the study

In order to prevent the dissemination of information, the hospitals were first divided into an intervention and a control group using simple random sampling (tap and line). The participants were selected by simple random sampling using the table of random numbers. Then, based on the hospitals, the patients were allocated to the intervention and control groups. During the study, one individual from the control group and two individuals from the intervention group were excluded due to transplantation. One of the control group participants was also excluded due to transfer to another hospital and one of the intervention group participants was removed due to intubation and hospitalization. Finally, data from 75 participants (38 in the control group and 37 in the intervention group) were analyzed (Fig. 1).

The inclusion criteria were having the ability to read and write, having the ability to speak and understand Persian, aging 18–65 years, being diagnosed with chronic renal failure by a nephrologist, and undergoing hemodialysis two or three times a week. The exclusion criteria were suffering from well-known psychiatric disorders such as severe depression, psychosis, bipolar disorder, and acute physical illnesses, being under a mechanical ventilator, having kidney transplants, and attending psychological training and interventions simultaneously.

### Data collection

The primary and secondary outcomes were hope and adherence to treatment, respectively. Data collection tools included a demographic information form, Snyder Hope Scale, End-stage renal disease adherence questionnaire (ESRD-AQ), laboratory evaluations such as Blood urea nitrogen (BUN), creatinine, phosphate, and potassium, and inter-dialytic weight gain.

Both groups completed the questionnaires of hope and adherence to treatment before and three months after the intervention. Similarly, the laboratory tests and inter-dialytic weight gain were measured before and three months after the intervention. In both intervention and control groups, BUN, creatinine, and phosphate levels were measured using similar kits (Man). A similar device (easy light) was also used to measure the potassium level. Furthermore, the patients' weights were measured using the calibrated scales in the centers. Finally, the inter-dialytic weight gain was calculated by subtracting the post-dialysis weight from the pre-dialysis weight in each session. The mean weight difference between the two consecutive dialysis sessions was then calculated as the final score.

Snyder Hope Scale was developed by Snyder et al. in 1991. In this 12-item questionnaire, the items could be responded via a five-point Likert scale ranging from completely disagree (score 1) to strongly agree (score 5), while the scoring was inverted for items 3, 5, 7, and 11. A higher score on the questionnaire indicated a higher hope in the respondents. Snyder et al. reported a negative correlation of 0.44 with the Beck Depression Inventory to assess the simultaneous correlation validity [27]. In addition, this scale showed a significant positive correlation with positive affect, optimism, life satisfaction, and self-esteem, but a significant negative correlation with anxiety and pessimism [28]. Snyder et al. calculated the reliability of the Hope Scale by the test–retest method after three (r = 0.85), eight (0.73), and 10 (0.82) weeks and approved the internal consistency through Cronbach's alpha coefficient (r = 0.84) [27].

ESRD-AQ was developed by Kim et al. in 2009. This questionnaire consisted of 46 questions/items divided into five sections. The first section pursued general information about the patients' history of ESRD and Renal Replacement Therapy (RRT) (5 items), while the remaining four sections asked about adherence to treatment (14 items), medications (9 items), fluid restrictions (10 items), and diet recommendations (8 items). These four final sections directly measured the adherence behaviors and the patients' knowledge and perceptions about the treatment. The ESRD-AQ items could be responded via Likert scales, multiple choices, or “yes/no” options. The scores of the questionnaire could range from zero to 1200, with higher scores representing higher adherence to treatment [29]. The item level content validities for the 46 items ranged from 0.86 to 1.00, which resulted in the average Item-level Content Validity Index (I-CVI) of 0.99. The fairly high level of CVI for each item implied that the content for the construct was adequately represented by the item. Additionally, strong test–retest stability existed across all items, with Intra-class Correlation Coefficients (ICCs) ranging from 0.83 to 1.00. Moreover, Phi correlations indicated that the self-reported adherence behaviors and perceptions were consistent across the two administrations of the ESRD-AQ [29].

### Intervention

The intervention included positive thinking training presented in eight 30-min sessions. The intervention was conducted by individual counseling on the patients' bedsides during their dialysis sessions. The educational content used in this study was selected from the book 'Positive Thinking and Applied Positivism' by Susan Quilliam, and the curriculum was designed accordingly [18]. In each session, practice was done on the topics discussed. The patients were also assigned assignments to perform at home. The assignments were reviewed at the beginning of each session. It should be noted that positive thinking training was provided by the first author in collaboration with a psychologist expert in positive thinking skills. The contents of the training included cognitive and behavioral skills of positive thinking, such as ways to moderate negative thoughts, institutionalizing positive thinking strategies, establishing constructive relationships, calming, bringing laughter to life, and building self-esteem (Table 1).

**Table 1:** The content of the positive thinking training program

Session 1	Expressing the goals and method of implementation of the program, the reason for selecting individuals, and familiarity with positive thinking
Session 2	Understanding how thinking and attitude are formed
Session 3	Familiarity with negative thoughts and ways of moderating them, positive thinking and its impact on people's health and life expectancy
Session 4	Teaching to be positive by challenging negative thoughts, changing mental images, and using constructive language and rethinking beliefs
Session 5	Teaching to be positive by institutionalizing positive thinking strategies in life, continuing to practice positive thoughts, opportunities for positive thinking through coping with problems we cannot solve
Session 6	Trying to live a positive way by building a positive relationship, building good relationships with those around, and love wholeheartedly
Session 7	Teaching to be positive by learning how to stop thinking, calming, changing attitudes towards eliminating the dos and don’ts, curbing negative thoughts, and struggling with negative thoughts
Session 8	Bringing laughter to life, building self-esteem, and developing a good sports habit

The control group participants received no interventions; they received only the routine training, including fluid restraint, diet, and medications, by the hemodialysis nurses.

To provide blindness, the statistician who performed the data analysis and the individual who was responsible for collecting the data were blind to the study groups.

### Data analysis

SPSS software, version 18 was used for data analysis. Descriptive statistics were used to describe the variables. Kolmogorov–Smirnov test was used for assessment of the normal distribution of the data. In addition, paired and independent t-tests were used to compare the means within and between groups, respectively. Chi-square test was also used to compare the two groups based on the demographic and clinical data.

## Results

The mean age of the participants was 51.1 ± 5.3 years. Most of the participants were female (52%), married (64%), and unemployed (82.6%) and had below diploma education levels (64%). More than half of the participants underwent hemodialysis three times a week (76%). The mean duration of hemodialysis treatment was 49.95 months. The results showed no significant difference between the two groups concerning the duration of the hemodialysis treatment (*p* = 0.5). There was also no significant difference between the two groups in terms of demographic and clinical variables (Table 2).

**Table 2:** Comparison of the two groups regarding demographic and clinical data

Variables	Intervention group (n = 37)	Control group (n = 38)	Chi-square	*P*-value
	n (%)	n (%)		
*Gender*				
Female	22 (29.3)	17 (22.6)	1.6	0.168
Male	15 (20)	21 (28)		
*Employment status*				
Employed	5 (6.6)	8 (10.6)	0.7	0.290
Unemployed	32 (42.6)	30 (40)		
*Marital status*				
Single	5 (6.6)	8 (10.6)	1.9	0.386
Married	23 (30.6)	25 (33.3)		
Widowed or divorced	9 (12)	5 (6.6)		
*Education level*				
High school	26 (34.6)	22 (29.3)	2.6	0.264
Diploma	5 (6.6)	11 (14.6)		
Academic	6 (8)	5 (6.6)		
*Frequency of hemodialysis per week*				
Twice a week	10 (13.3)	8 (10.6)	0.3	0.597
Three times a week	27 (36)	30 (40)		

Before the intervention, the two groups were homogeneous regarding the mean scores of ESRD-AQ, laboratory tests, and hope. After the intervention, however, the intervention group showed a significant improvement in the mean score of treatment adherence, BUN level, phosphate level, and mean inter-dialytic weight gain compared to the control group. The mean score of hope was also higher in the intervention group (42.1 ± 6.1) compared to the control group (38.7 ± 6.5) after the intervention. However, no statistically significant difference was found between the two groups in term of potassium and creatinine levels after the intervention (Table 3).

**Table 3:** Comparison of the two groups regarding treatment adherence, laboratory tests, inter-dialytic weight gain, and hope before and after the intervention

Dimensions	Group	Pre-test		Post-test		*p*-Value*
		Mean	SD	Mean	SD	
Treatment adherence	Intervention	1037.8	103.5	1070.2	80.1	0.013
	Control	1013.8	117.9	1018.4	105.3	0.772
	**p-Value	0.352		0.019		
Blood urea nitrogen	Intervention	56.3	13.9	53.0	14.1	0.008
	Control	57.8	12.1	59.3	12.1	0.180
	**p-Value	0.611		0.045		
Creatinine	Intervention	5.5	0.7	5.3	0.6	0.166
	Control	5.4	0.5	5.3	0.6	0.270
	**p-Value	0.503		0.680		
Potassium	Intervention	5.07	0.5	4.9	0.4	0.195
	Control	5.1	0.4	5	0.4	0.243
	** p-Value	0.768		0.589		
Phosphorus	Intervention	5.6	0.9	5.2	0.7	0.012
	Control	5.8	0.9	5.6	0.8	0.093
	**p-Value	0.206		0.049		
Inter-dialytic weight gain	Intervention	2.1	0.7	1.9	0.7	0.019
	Control	2.3	0.5	2.2	0.6	0.428
	**p-Value	0.090		0.031		
Hope score	Intervention	39.6	5.5	42.1	6.1	0.001
	Control	38.9	4.7	38.7	6.5	0.770
	**p-Value	0.542		0.024		

*Paired t-test

**Independent t-test

## Discussion

The results of the present study indicated that eight sessions of positive thinking training along with assignments and practice promoted adherence to the treatment and hope among the hemodialysis patients. Previous studies have also revealed the role of individual attitudes and positive thinking in adherence to treatment in chronic diseases [30–33]. For instance, the results of a study conducted on African Americans demonstrated that the positive attitudes of patients with diabetes predicted their adherence to the medication regimen [30]. In addition, induction of positive affect and self-affirmation increased physical activity in patients after percutaneous coronary intervention. These were induced through training, emphasis on positive thoughts, and creation of positive self-esteem through telephone calls and unexpected small mail gifts [32].

The present study results revealed an improvement in weight control as well as in some laboratory tests, such as BUN and phosphate levels, after the intervention. However, no significant change was observed in potassium and creatinine tests. Serum levels of phosphate, potassium, creatinine, and BUN indicate a patient's adherence to diet and medication, and weight gain between two dialysis sessions shows the patient's adherence to fluid regimens [34]. Therefore, it can be argued that positive thinking training promoted adherence to fluid restriction and some of the consequences of adherence to diet and medication among the patients. In the same vein, the findings of the previous studies approved the effects of psycho-educational interventions on improvement of adherence to treatment in hemodialysis patients. As an instance, using Benson's relaxation technique for eight weeks could improve the BUN and phosphorus levels of hemodialysis patients and reduced their inter-dialytic weight gain [35]. In another study, psychological interventions, including group discussions, over five weeks led to weight loss between the two dialysis sessions [36]. On the contrary, an empowerment program involving individual and group counselling did not change the laboratory results of hemodialysis patients, except for hemoglobin and hematocrit levels [37]. A possible explanation for the lack of significant change in the potassium levels among the patients in the current investigation is that it was within a high normal range at the baseline. The intervention has had a minimal effect on the potassium level and it was maintained within the normal range at the end of the study. Furthermore, observation of no changes in the creatinine levels could be related to the fact that the long-term effects of the intervention were not examined. Hence, it is suggested that in future studies, people practice positive thinking for a longer period of time and then the results of the study are evaluated. Most of the patients in the present study were middle-aged and married. Therefore, they received sufficient family support. However, most of them were unemployed and underwent hemodialysis three times a week. These could make these patients more vulnerable to psychological problems, including hopelessness. Previous studies have also raised the possibility of hopelessness among hemodialysis patients [25, 38].

The findings of the present study showed that positive thinking training increased hope among the patients. Prior studies have also revealed the effects of positive thinking on psychological variables of individuals in different groups. In this regard, positive thinking training improved hope among elderly individuals [39]. In addition, a brief positive psychology intervention, including watching a video containing positive thoughts and feelings and three positive thinking exercises, increased the hope of young people in a city in Greece [40]. In the same line, a positive thinking intervention with the Islamic approach, including seven sessions of group therapy, increased the hope of patients with multiple sclerosis [41]. Besides, training based on positive thinking proposed by Martin Seligman increased hope in patients with thalassemia [22].

In this study, due to the intimate and close relationships among the patients undergoing hemodialysis, it was not possible to randomly assign the participants to the two groups. Thus, random allocation was done based on the hospitals. In addition, the participants were aware that they belonged to the control or the intervention group. These two limitations could reduce the generalizability of the findings. However, the use of face-to-face training at the time of hemodialysis was favorable for the patients in the intervention group and made them more willing to continue the study. Other strengths of the study included the use of a two-group, pre-post design, practices in each training session, and presentation of assignments in each session.

Given the lack of research on the role of positive thinking in the hope of patients undergoing hemodialysis, it can be concluded from the results of the present study and the above-mentioned studies that interventions inducing positive thinking and motivation could increase hope among patients under hemodialysis. Therefore, because of its cost-effectiveness, this method can be used to increase these patients' hope.

## Conclusion

According to the results of the current study, positive thinking interventions could improve hope and increase adherence to treatment among the patients under hemodialysis probably by reducing hopelessness, emphasizing the positive aspects of patients' lives, and training strategies for accepting the realities and enhancing their physical and mental abilities. Therefore, the use of positive thinking could be recommended to improve adherence to treatment and hope in patients undergoing hemodialysis. By conducting further studies in this area, positive thinking training can be considered a feasible, low-cost, and safe non-pharmacological intervention as a complement to psychological and nursing interventions to improve treatment adherence and hope among patients undergoing hemodialysis.

## Data Availability

The datasets used during the current study available from the first author on reasonable request.

## Abbreviations

ESRD: End stage renal disease
ESRD-AQ: End-stage renal diseaise adherence questionnaire
BUN: Blood urea nitrogen
SPSS: Statistical package for the social sciences
